# Traumatic and Atraumatic Rotator Cuff Tears Have the Same Rates of Healing

**DOI:** 10.1016/j.asmr.2023.100867

**Published:** 2024-02-13

**Authors:** Alberto Guevara-Alvarez, Edwin A. Valencia-Ramon, Hugo Bothorel, Philippe Collin, Jeanni Zbinden, Alberto Guizzi, Alexandre Lädermann

**Affiliations:** a Instituto de Hombro IDH, Hospital Angeles Querétaro, Querétaro, México; b Research Department, La Tour Hospital, Meyrin, Switzerland; c American Hospital of Paris, Neuilly-sur-Seine, France; d Division of Orthopaedics and Trauma Surgery, La Tour Hospital, Meyrin, Switzerland; e Department of Medical and Surgical Specialties, Radiological Sciences, and Public Health, University of Brescia, Brescia (BS), Italy; f Faculty of Medicine, University of Geneva, Geneva, Switzerland; g Division of Orthopaedics and Trauma Surgery, Department of Surgery, Geneva University Hospitals, Geneva, Switzerland

## Abstract

**Background:**

To examine whether traumatic rotator cuff repairs (RCRs) differ in postoperative rotator cuff tendon integrity and functional outcomes from degenerative RCRs.

**Methods:**

RCRs performed by a single surgeon were retrospectively identified. The inclusion criteria were repairable Goutallier grades 0 to 2 full-thickness rotator cuff tears. Demographic and clinical data as well as radiological results were compared. A multivariate logistic regression of the of patient acceptable symptom state for American Shoulder and Elbow Surgeons (ASES) score was performed to evaluate whether the origin of tear led to a different relative risk (RR) independently from tear and surgical characteristics.

**Results:**

A total of 616 consecutive shoulders (304 traumatic and 312 degenerative) were finally included. Traumatic ruptures presented a greater distribution of male (72% vs 51%, *P* < .001) and younger patients (53 vs 57 years, *P* < .001), as well as earlier onset of symptoms (3 vs 15 months, *P* < .001), reduced range of motion in preoperative assessment for forward elevation (130° vs 150°, *P* < .001), and slightly greater preoperative ASES (46.5 ± 19.7 vs 50.0 ± 18.0, *P* = .022) and Constant (47.0 ± 20.2 vs 52.0 ± 18.9, *P* = .001) scores. Degenerative tears presented a lower proportion of grade 3 tendon coronal retraction (11% vs 18%, *P* = .031). Postoperative tendon integrity at 6 months was comparable for both groups, predominantly Sugaya types 1 and 2 (91% traumatic; 92% degenerative, *P* = .371). Both groups exhibited favorable outcomes in range of motion and postoperative functional scores at last follow-up. The multivariate regression confirmed that the tear origin was not significantly associated with patient acceptable symptom state achievement (*P* = .201) but rather with greater preoperative ASES score (RR, 1.01), men (RR, 1.16) and workers’ compensation (RR, 0.65) (*P* < .05).

**Conclusions:**

Traumatic cases were frequent, involved younger patients, more frequently affected the anterior rotator cuff, and were associated with more severe tendon retraction. Traumatic and degenerative RCRs lead to comparable clinical and radiologic results.

**Level of Evidence:**

Level III, retrospective comparative study.

The etiology of rotator cuff tears is multiple but predominantly composed of 2 major types: traumatic and degenerative. The former is not simply the acute failure of a degenerative tendon; the literature suggests that traumatic tears signify a unique pathologic condition.^1^ Traumatic tears are considered to present with a greater prevalence in younger patients, with more anterior involvement and less muscle atrophy, fatty degeneration, and tendon retraction.^1^, ^2^, ^3^ Therefore, due to the better structural condition of the tendon, it is reasonable to assume a superior healing rate and clinical results in the traumatic setting.

The literature comparing these 2 types of rotator cuff repairs (RCRs) is sparse.^4^, ^5^, ^6^, ^7^, ^8^, ^9^, ^10^, ^11^ Most studies are small cohorts with unreliable and loose criteria distinguishing traumatic from degenerative tears.^4^^,^^5^^,^^8^^,^^10^ Furthermore, the results of these studies are contradictory, including those that have concluded similar outcomes between the 2 types of injuries,^9^^,^^11^^,^^12^ and others that have reported superior outcomes in traumatic settings.^6^^,^^7^ Consequently, greater functional results and healing rates of traumatic RCRs have yet to be proven.

The purpose of this study was to examine whether traumatic RCR differs in postoperative rotator cuff tendon integrity and functional outcomes from degenerative RCR. The hypothesis was that traumatic tears would have superior clinical and radiologic outcomes to degenerative tears.

## Methods

### Study Design and Patient Selection

All patients with an RCR performed by the senior author (A.L.) between January 2015 and December 2020 were considered potentially eligible for inclusion in this retrospective comparative study. Data were extracted from a prospective RCR database (Follow Health, Rennes, France), the use of which had been approved (Association des médecins du canton de Genève et société médicale #12-26). All patients have been included in various research protocols approved by the local ethical committee (CCER 2015-15 trial registration number NCT02725346, CCER 2016-00818 trial registration number NCT02943005, 2019-02076 trial registration number NCT04321005)^13^ and provided written informed consent for their participation and the use of their data and images for research and publishing purposes. The inclusion criteria involved all patients presenting full-thickness tears on magnetic resonance imaging with or without arthrography with the potential for complete arthroscopic RCR. Only repairable tears of Goutallier grades 0 to 2 were included,^14^ using a modified 2-grade scale of the Goutallier classification according to the study by Sheean et al.^15^ The 2-grade scale is outlined as grade 1 (Goutallier 0, 1 and 2) and grade 2 (Goutallier 3 and 4), with the standard 5-grade scale having a poor-to-moderate agreement.^16^ These patients were divided into 2 groups: a traumatic group and a degenerative group. In Switzerland, an accident is defined by strict medical (demographic, clinical, factorial, radiologic, and intraoperative) criteria, based not on medical or patient judgment but solely on a legal and neutral analysis of these criteria.^17^^,^^18^ The following 5 conditions must be satisfied: the trauma must result from an external agent (cannot be self-inflicted), be harmful, sudden, involuntary, and extraordinary (cannot be part of a usual activity).^19^ Pre-existing pathologies with an acute decompensation (i.e., acute on chronic tears) were considered as degenerative tears. Patients were excluded who presented a history of (1) previous shoulder surgery; (2) chronic dislocation; (3) preoperative infection; (4) rotator cuff arthropathy with glenohumeral osteoarthritis and superior migration of the humeral head; (5) psychiatric problems that precluded informed consent or inability to read or write; (6) fatty infiltration of grade 3 or 4^14^; (7) unusual tears including Fosbury,^20^ frayed upper-edge subscapularis lesion with impingement,^21^ subscapularis abrasion from the middle glenohumeral ligament (SAM lesion),^22^ or musculotendinous junction lesions^23^; (8) partial tears^15^; (9) follow-up time of less than 2 years; and (10) incomplete documentation.

### Study Variables

The primary outcome was rotator cuff tendon healing determined by ultrasound scan^24^ at the 6-month^25^^,^^26^ postoperative follow-up. Secondary outcomes included pain and various functional scores at 6 months and last follow-up. In addition, the following baseline characteristics were assessed: age, sex, shoulder side, limb dominance, multiple comorbidities such as diabetes mellitus, hypertension, hypercholesterolemia, tobacco use, delay between symptom onset and surgical treatment, workers’ compensation status, shoulder range of motion (ROM), and the etiology, type, and retraction of tears.

### Surgical Technique

A consistent operative technique was used during the study period. The tears were described according to classifications of Collin et al.^27^ and Patte.^28^ Previously reported single- or double-row techniques were used,^29^^,^^30^ depending on the classification and characteristics of the tears (L, U, V-shapes).^31^ Tenodesis or tenotomy of the long head of the biceps was systematically performed. Adjuvant acromioplasty was performed in patients who had radiographic signs of dynamic impingement or to improve visualization,^32^, ^33^, ^34^ and resection of the distal section of the clavicle was performed when pain was elicited by palpation of the acromioclavicular joint. At the end of the intervention, all RCRs were complete and “watertight,” with adequate restoration of the tendons to their footprints.

### Postoperative Rehabilitation

During their hospitalization, all patients received postsurgical recommendations tailored to the type of RCR.^35^ Patients having received anterior RCRs were immobilized in a sling during 4 to 6 weeks, depending on the intraoperative difficulty of reduction and the subjective quality of the RCR. Patients with massive posterosuperior RCRs were immobilized in an abduction pillow sling for the same period of time according to the same criteria.^36^ No sling was recommended to patients with superior small-to-large RCRs.^37^ For the latter group, active-assisted mobilization was proposed during the first 4 postoperative weeks. These patients were prohibited from performing active abduction–elevation (only passive-assisted abduction-elevation was permitted). For all RCRs, exercise and light sports were authorized after 6 weeks and 2 months, respectively. A gentle strengthening program was permitted after 3 postoperative months.

### Clinical and Radiologic Examination

Patient outcomes were recorded preoperatively and at 6 months and at a minimum of 2 years postoperatively with the pain on a visual analog scale (VAS), the American Shoulder and Elbow Surgeons (ASES),^38^ and Constant^39^ scores, the Simple Shoulder Value (SSV),^40^ and ROM. Patients completed the ASES, pain, and SSV scores, along with the subjective components of the Constant score, using tablets equipped with the Follow Health software while waiting in the reception area. ROM and strength measurements were conducted by independent evaluators who were not part of the current study, under the supervision of the senior author (A.L.), ensuring the reproducibility of the results. Active anterior elevation and external rotation in 0° of abduction were assessed with a goniometer, the patient being in the upright position. Internal rotation behind the back was estimated to the nearest spinal level and noted on a 20-level scale ranging from 0 points (thigh) to 19 points (T1 thoracic spine). At 6 months, tendon repair integrity according to the Sugaya classification was assessed by means of ultrasound by the senior author (A.L.) according to a validated protocol.^25^^,^^26^^,^^41^

### Statistical Analyses

For baseline characteristics, variables were reported as proportions or mean ± standard deviation, median and range. Shapiro-Wilk tests were used to assess the normality of distributions. For non-Gaussian continuous data, differences between groups were evaluated using Wilcoxon rank-sum tests (Mann-Whitney *U* test). For Gaussian continuous data, differences between groups were assessed using unpaired Student *t*-test. For categorical data, differences between groups were evaluated using the χ^2^ or Fisher exact test where appropriate. The minimal clinically important difference as well as patient acceptable symptom state (PASS) for the principal outcome of the study were used to better evaluate the proportion of patients who were clinically improved after surgery (ASES score, 11.1 and 86.7 points, respectively).^42^ A multivariate logistic regression of the PASS for the ASES score was performed to identify whether the traumatic or degenerative group was associated with better postoperative outcomes independently from patient pre- or intraoperative characteristics. Given the nonrarity of this event (>10%-20%), a modified Poisson regression approach was also used to provide relative risks^43^ and avoid the misinterpretation of odds ratios.

Statistical analyses were performed using R, version 3.6.2 (R Foundation for Statistical Computing, Vienna, Austria). *P* values < .05 were considered statistically significant.

An a priori sample size calculation was performed to ensure a sufficient statistical power for the multivariate logistic regression. According to Patel et al.,^44^ 35% of the patients arthroscopically operated for RCRs failed to achieve the PASS of the ASES score at 1 postoperative year. Following the 10 events per variable, 343 patients would be required in the model (thus including 120 patients who failed to reach the PASS) to have a sufficient statistical power with 12 potential predictors included.

## Results

**Fig 1:**
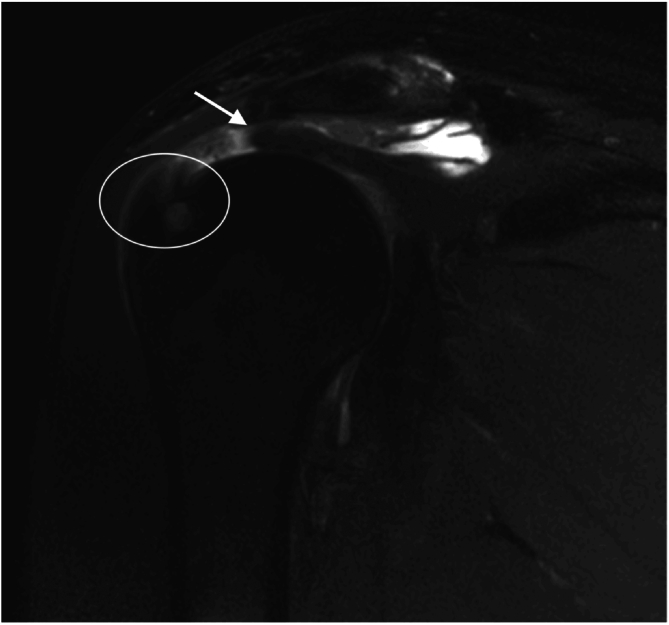
Right shoulder MRI, T2-weighted fat-saturated coronal view of a traumatic tendon shortening 2 months after a fall while hiking. Observe the residual stump at the greater tuberosity^45^ (B2^31^ (midsubstance) lesions) (circle). The supraspinatus tendon has shortened (arrow). (MRI, magnetic resonance imaging.)

A total of 647 shoulders satisfied the inclusion criteria in the time frame selected, and 39 (6.0%) were excluded (Fig 1).^45^ The final cohort comprised 616 shoulders, of which 312 (50.6%) presented a degenerative injury and 304 (50.4%) were identified as traumatic. Both groups were comparable in terms of hypertension (*P* = .481), hypercholesterolemia (*P* = .421), diabetes (*P* = .848), and smoking status (*P* = .138) (Table 1). However, the traumatic group was characterized by a greater distribution of male patients (72% vs 51%) and younger patients (53 ± 10 years vs 57 ± 9 years) compared with the degenerative group (*P* < .001). The onset of symptoms before surgery significantly differed between groups, with a median of 3 months for the traumatic group compared with 15 months for the degenerative group (*P* < .001). In the traumatic group, 81 patients (25.8%) were operated in the month following the onset of the symptoms.

**Table 1:** Patient Characteristics

	Degenerative RCTs (n = 304)			Traumatic RCTs (n = 312)			*P* Value
	N (%)	Median	(Range)	Mean ± SD	Median	(Range)	
	Mean ± SD						
Age, y	57.1 ± 9.3	57.0	(22.0 - 79.0)	53.0 ± 9.5	52.0	(24.0 - 77.0)	**≤.001**
BMI	26.3 ± 7.8	25.6	(17.1 - 132.5)	26.1 ± 4.2	25.5	(18.1 - 40.4)	.514
Weight, kg	75.6 ± 15.4	75.0	(42.0 - 145.0)	78.8 ± 14.4	78.0	(44.0 - 132.0)	**.005**
Height, cm	170.4 ± 11.0	172.0	(65.0 - 192.0)	173.7 ± 8.6	175.0	(150.0 - 200.0)	**≤.001**
Symptoms onset before surgery, mo	23.4 ± 25.8	15.0	(0.0 - 225.0)	5.7 ± 9.2	3.0	(0.0 - 84.0)	**≤.001**
Male sex	155 (51.0%)			225 (72.1%)			**≤.001**
Hypertension	45 (18.0%)			41 (15.6%)			.481
Hypercholesterolemia	34 (13.6%)			29 (11.1%)			.421
Diabetes	13 (5.2%)			15 (5.7%)			.848
Smokers	76 (29.9%)			64 (23.9%)			.138
Workers' compensation	81 (26.6%)			93 (29.8%)			.421
Dominant arm	221 (72.7%)			213 (68.3%)			.251
Acromioplasty	271 (89.1%)			225 (72.1%)			**≤.001**
Tear type							**.006**
Collin classification: A, B, C, D, E	13.8%, 3.0%, 13.8%, 25.7%, 1.0%			7.4%, 5.8%, 16.7%, 27.2%, 0.6%			
Isolated tear: infra, supra, subscap	0.0%, 37.5%, 5.3%			0.3%, 30.8%, 11.2%			
Tendon retraction (Patte classification)							**.031**
1	163 (53.6%)			142 (45.5%)			
2	107(35.2%)			114 (36.5%)			
3	34 (11.2%)			56 (17.9%)			
Clavicular resection	67 (22.0%)			36 (11.5%)			**.001**
LHB or capsular procedure							**≤.001**
None	24 (7.9%)			26 (8.3%)			
LHB tenodesis	141 (46.4%)			194 (62.2%)			
LHB tenotomy	139 (45.7%)			92 (29.5%)			
Suture technique							.809
Single row	162 (53.3%)			163 (52.2%)			
Double row	142 (46.7%)			149 (47.8%)			

NOTE. Values in bold indicate significant *P* values (*P* < .05).

BMI, body mass index; Infra, infraspinatus; LHB, long head of the biceps tendon; SD, standard deviation; Subscap, subscapularis; Supra, supraspinatus.

According to the morphology of the tear, Collin type D was the more frequent variety in both groups (27.2% and 25.7%), despite some discrepancies in other rupture patterns. Traumatic tears more frequently involved the subscapularis with Collin type B (5.8% vs 3.0%) and type C injuries (16.7% vs 13.8%) (Table 1), as well as isolated subscapularis tears (11.2% vs 5.3%) (*P* = .006). In contrast, degenerative RCRs presented a greater prevalence of Collin type A (13.8% vs 7.4%), or isolated supraspinatus tears (37.5% vs 30.8%) (*P* = .006). Retraction of Patte type III was rarer among degenerative RCRs, with 11.2% as opposed to 17.9% among traumatic cases (*P* = .031). The preoperative clinical presentation of patients with traumatic tears was noteworthy for reduced ROM in forward flexion (128° vs 149°, *P* < .001), external rotation (38° vs 45°, *P* < .001), and internal rotation (7 ± 4 vs 8 ± 4, *P* < .001) (Table 2).

**Table 2:** Pre- and Postoperative Outcomes

	Degenerative RCTs (n = 304)				Traumatic RCTs (n = 312)				*P* Value
	N	(%)	Median	(Range)	Mean	±SD	Median	(Range)	
	Mean	±SD							
Forward flexion, °									
Baseline/preoperative	149	± 31	160	(0- 180)	128	± 50	150	(0- 180)	**≤.001**
6 mo	151	± 23	160	(70- 180)	148	± 22	150	(60- 180)	.103
Last follow-up	162	± 22	170	(45- 180)	162	± 22	170	(45- 180)	.941
External rotation (ER, °)									
Baseline/preoperative	45	± 19	40	(0- 90)	38	± 22	40	(0- 90)	**≤.001**
6 mo	41	± 18	40	(0- 130)	37	± 18	40	(0- 90)	**.041**
Last follow-up	62	± 25	60	(10- 160)	60	± 26	60	(0- 150)	.601
Internal rotation (IR, score)									
Baseline/preoperative	8	± 4	8	(1- 13)	7	± 4	8	(1- 13)	**≤.001**
6 mo	8	± 4	8	(1- 13)	8	± 4	8	(1-13)	.713
Last follow-up	9	± 4	8	(1- 13)	9	± 4	8	(1- 13)	.799
Pain on VAS									
Baseline/preoperative	59.0	± 17.8	60.0	(10.0- 100.0)	56.5	± 18.2	57.0	(0.0- 100.0)	.103
6 mo	21.1	± 19.0	17.0	(0.0- 80.0)	18.1	± 16.3	13.0	(0.0- 70.0)	.120
Last follow-up	13.7	± 19.3	3.0	(0.0- 90.0)	13.3	± 21.0	7.0	(0.0- 165.0)	.744
ASES score									
Baseline/preoperative	50.0	± 18.0	50.0	(10.0- 88.0)	46.5	± 19.7	48.0	(5.0- 90.0)	**0.022**
6 mo	74.3	± 15.5	77.0	(0.0- 100.0)	76.1	± 14.2	78.0	(0.0- 100.0)	.251
Last follow-up	85.9	± 16.3	91.0	(26.0- 100.0)	87.6	± 15.6	93.0	(30.0- 100.0)	.190
MCID or greater (11.1 points)	270	(88.8%)			285	(91.3%)			.293
PASS or greater (86.7 points)	189	(62.2%)			214	(68.6%)			.078
Constant score									
Baseline/preoperative	52.0	± 18.9	55.0	(7.0- 90.0)	47.0	± 20.2	49.0	(2.0- 93.0)	**.001**
6 mo	71.5	± 15.6	75.0	(0.0- 100.0)	73.7	± 14.0	76.0	(19.0- 100.0)	.128
Last follow-up	79.0	± 16.0	82.0	(25.0- 100.0)	80.9	± 16.6	84.0	(17.0- 100.0)	**.043**
SSV									
Baseline/preoperative	45.7	± 21.5	50.0	(0.0- 90.0)	45.6	± 22.9	50.0	(0.0- 100.0)	.964
6 mo	75.2	± 17.0	80.0	(10.0- 100.0)	76.8	± 15.5	80.0	(30.0- 100.0)	.345
Last follow-up	87.1	± 15.6	90.0	(0.0- 100.0)	86.2	± 19.7	90.0	(0.0- 100.0)	.399
Postoperative tendon healing									.371
Type 1	233	(76.6%)			236	(75.6%)			
Type 2	47	(15.5%)			48	(15.4%)			
Type 3	14	(4.6%)			19	(6.1%)			
Type 4	7	(2.3%)			6	(1.9%)			
Type 5	3	(1.0%)			3	(1.0%)			
Postoperative work return∗									.136
No	26	(10.6%)			35	(12.3%)			
Yes	207	(84.1%)			222	(78.2%)			
Planified	13	(5.3%)			27	(9.5%)			
Patient satisfaction at last follow-up	274	(90.1%)			284	(91.0%)			.783

NOTE. Values in bold indicate significant *P* values (*P* < .05).

ASES, American Shoulder and Elbow Surgeons; ER, external rotation; IR, internal rotation; MCID, minimum clinically important difference; PASS, patient acceptable symptom state; RCT, randomized controlled trial; SSV, Simple Shoulder Value; VAS, visual analog scale.

∗ Evaluated at 6 months (retired patients excluded).

Suture technique did not differ between groups, with 47% to 48% of the patients operated with a double-row technique in both groups (*P* = .809). Acromioplasty (89.1% vs 72.1%, *P* < .001) and clavicle resection (22.0% vs 11.5%, *P* < .001) were more readily performed in the degenerative group. Biceps tenodesis was implemented more frequently in patients with traumatic injuries (62.2% vs 46.4%, *P* < .001), whereas tenotomy was more often performed among degenerative patients (45.4% vs 29.5%, *P* < .001).

Postoperative tendon integrity at 6 months was comparable for both groups, of predominantly Sugaya types 1 and 2 (91% traumatic; 92.1% degenerative) (Table 2). The majority of patients in both groups (88%-89%) returned to work or planned to return the days following the 6 months’ control. No difference in the postoperative complications was observed (Table 3). At a last follow-up of 3.9 ± 1.8 years (range, 2-8 years) for the degenerative group and 3.7 ± 1.8 years (min-max, 1-8) (*P* = .188), both cohorts significantly improved, and the preoperative ROM differences disappeared postoperatively. A slightly greater Constant score was reported by traumatic group (80.9 ± 16.6 vs 79.0 ± 16.0, *P* = .043), which tended to comprise a greater proportion of patients reaching the PASS for the ASES score (69% vs 62%, *P* = .078) (Table 2). Although the traumatic group tended to have greater odds of reaching this PASS in the univariate analysis (odds ratio, 1.34; *P* = .084), the multivariate regression confirmed the absence of a significant association when deleting confounding effects with tear or surgical characteristics (odds ratio, 1.28; *P* = .201) (Table 4). The PASS achievement was, however, significantly associated with greater preoperative ASES score (relative risk [RR], 1.01; *P* < .001), men (RR, 1.16; *P* = .020), and workers’ compensation (RR, 0.65; *P* < .001).

**Table 3:** Postoperative Adverse Events

	Degenerative RCTs (n = 304)		Traumatic RCTs (n = 312)		*P* Value
	N	(%)	N	(%)	
New traumatic event	3	(1.0%)	2	(0.6%)	.671
Rotator cuff repair	**2**	(0.7%)	**2**∗	(0.6%)	
Nonhealing at 6 months or retear	14	(4.6%)	11	(3.5%)	.579
Rotator cuff repair	**0**	(0.0%)	**2**∗	(0.6%)	
Reverse shoulder arthroplasty	**1**	(0.3%)	**1**	(0.3%)	
Complications with short-term impact	8	(2.6%)	6	(1.9%)	.618
Infection	0	(0.0%)	1	(0.3%)	
Rotator cuff repair	**0**	(0.0%)	**1**	(0.3%)	
Frozen shoulder	4	(1.3%)	4	(1.3%)	
AC arthropathy	1	(0.3%)	1	(0.3%)	
Reoperation	**1**	(0.3%)	**0**	(0.0%)	
Complications with long-term impact	3	(1.0%)	0	(0.0%)	–
Glenohumeral osteoarthrosis	3	(1.0%)	0	(0.0%)	

NOTE. Values in bold indicate the surgical treatment of the complication.

AC, acromioclavicular; RCT, randomized controlled trial.

∗ Two patients had a nonhealing of the tendon at 6 months and a new traumatic event.

**Table 4:** Uni- and Multivariate Logistic Regression Analyses of PASS Achievement for the ASES Score

		Univariate			Multivariate			
		OR	95% CI	*P* Value	OR	95% CI	RR∗	*P* Value
RCT group				–				–
Degenerative		REF			REF		REF	
Traumatic		1.34	(0.96-1.88)	.084	1.28	(0.85-1.92)	1.08	.201
Follow-up, y		1.07	(0.98-1.18)	.144	1.05	(0.94- 1.16)	1.01	.393
Preoperative ASES score		1.02	(1.01-1.03)	**≤.001**	1.02	(1.01-1.03)	1.01	**≤.001**
Symptoms onset before surgery, mo		1.00	(0.99-1.00)	.310	1.00	(0.99-1.01)	1.00	.544
Age		1.00	(0.99- 1.02)	.610	1.00	(0.98-1.02)	1.00	.811
Sex								–
Women		REF			REF		REF	
Men		1.55	(1.11- 2.18)	**.011**	1.62	(1.11- 2.38)	1.16	**.020**
Acromioplasty		0.90	(0.58- 1.36)	.613	1.02	(0.62- 1.64)	1.01	.891
Workers' compensation		0.32	(0.22- 0.46)	**≤.001**	0.32	(0.21- 0.47)	0.65	**≤.001**
Distal clavicle resection		0.80	(0.52- 1.24)	.310	0.93	(0.57- 1.52)	0.98	.791
Patte classification								
1		REF			REF		REF	
2		1.14	(0.79- 1.64)	.500	1.06	(0.70- 1.58)	1.01	.814
3		0.98	(0.60- 1.61)	.920	0.98	(0.56- 1.73)	0.99	.912
LHB procedure								
None		REF			REF		REF	
Tenodesis		1.04	(0.54- 1.92)	.904	1.05	(0.52- 2.04)	1.01	.949
Tenotomy		0.90	(0.46- 1.69)	.737	0.87	(0.42- 1.72)	0.95	.656

ASES, American Shoulder and Elbow Surgeons; β, regression coefficient; CI, confidence interval; LHB, long head of the biceps tendon; OR, odds ratio; PASS, patient acceptable symptom state; RCT, randomized controlled trial; REF, reference; RR, relative risk.

∗ Obtained through a modified Poisson regression approach.

## Discussion

The results of the present study provide no evidence to support a difference between traumatic and degenerative RCRs in tendon healing rate at the 6-month follow-up and clinical scores at a minimum of 2 years, despite precipitated intervention in the traumatic context. Therefore, the posited hypothesis was not confirmed. Tendon retraction and quality, muscle edema, pseudo-fatty infiltration, and timing of surgery may possibly contribute to these findings. These results are important because they challenge a contrary preconception. The conclusion is that traumatic and degenerative RCRs both lead to equally satisfactory results and thus merit equal attention.

### Patient Acceptable Symptom State

Interestingly, traumatic RCRs tended to have slightly greater postoperative outcomes compared with degenerative RCRs, although they presented slightly worse function preoperatively, as underlined by the ROM and patient-reported outcome measures. The analyses revealed, however, that traumatic and degenerative RCRs differed in terms of sex distribution, and other pre- or intraoperative characteristics, which could also independently have an impact on the outcomes. After we deleted the confounding effects between variables, this tendency was no longer true. Our adjusted results furthermore demonstrated that men had a 16% greater chance of reaching the PASS for the ASES score compared with women. Since men are more affected by traumatic tears in our studied cohort, such association could explain why traumatic cases reached the PASS in a greater proportion.

### Tendon Retraction

Degenerative tears are believed to present more significant retraction compared with traumatic ones,^2^ a factor that would negatively influence functional outcomes and healing.^46^ Conversely, a greater number of retractions of Patte grade 2 to 3 were observed in the traumatic group. Several explanations may clarify this finding. First, there is no rationale regarding retraction as the hallmark of degenerative tears. Experimental studies have shown that all muscles retract significantly immediately after a tendon release.^47^ Second, the previous studies that have observed greater retraction among degenerative tears were based on small sample sizes^2^ and may not be representative. Lastly, in the frontal plane, traumatic tears generally involve the tendon itself^48^ (B2 lesions, also known as midsubstance tears)^31^ compared with degenerative tears that generally correspond to a detachment from the bone (B1 lesions).^31^ Thus, the remaining stump on the bone in traumatic tears participates in tendon shortening, thereby mimicking retraction (Fig 1).

### Tendon Quality

**Fig 2:**
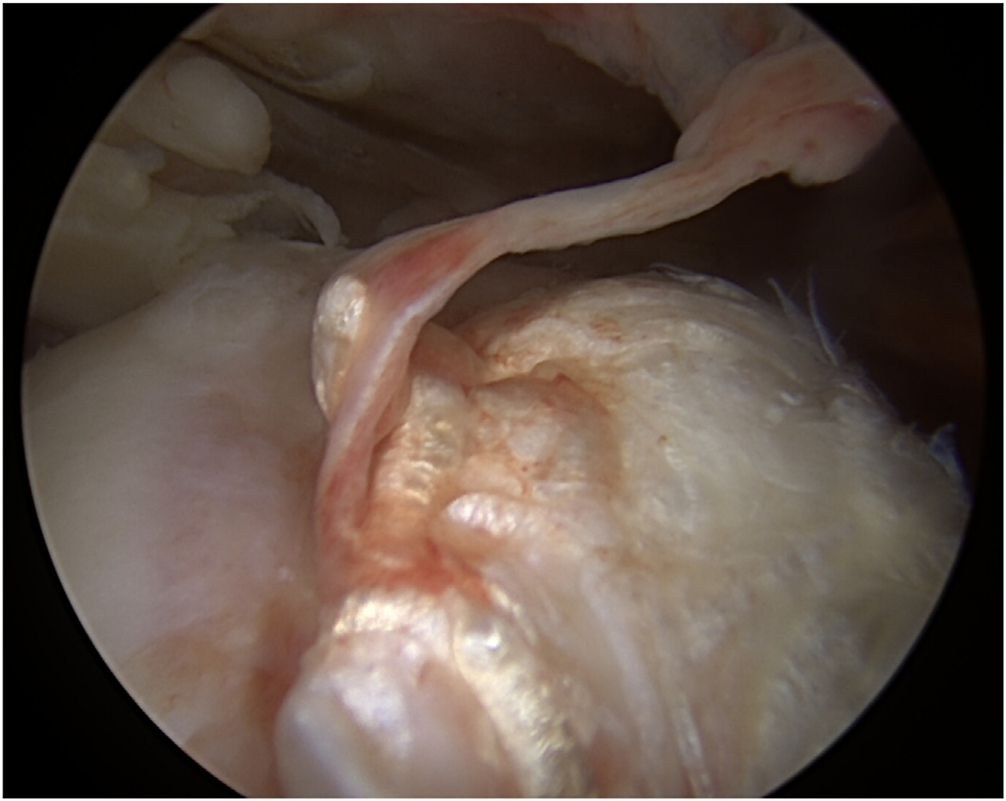
Arthroscopic characteristics of traumatic tendon tears, viewing posterolaterally from a right shoulder. Observe the stump of the remaining posterosuperior cuff on the greater tuberosity, tendon shredding, petechiae, and wavy form of torn rotator cuff edge, classical signs of traumatic tears.^45^

**Fig 3:**
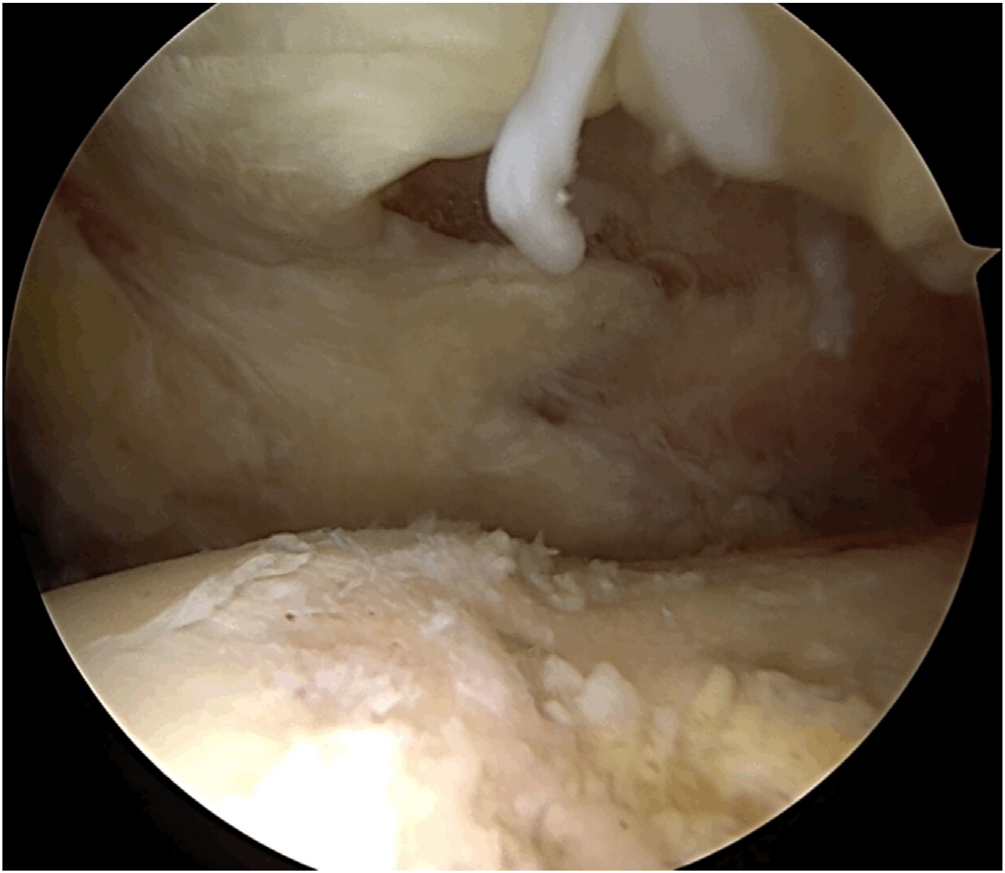
Arthroscopic characteristic of a right degenerative tear, viewing from a posterolateral portal. The tendon is thinner and rounded, and there is no visible stump in the tuberosity.

Trauma can lead to tendon shredding (Fig 2).^45^ In contrast, chronic wear is generally characterized by thinner but more regular tendons (Fig 3).^49^^,^^50^ There is no evidence that such shredding is more favorable to healing than thinning.

### Muscle Edema, Muscular Fibrosis, and Pseudo-fatty Infiltration

Traumatic reparable tears may be associated with muscle edema,^31^ a phenomenon involving the deterioration of muscle fiber bundles and, as early as 3 weeks, development of intramuscular fibrosis.^47^ Muscle edema is therefore not anodyne and may explain the difficulty in reducing tears only some months after the trauma, compromising final results.^4^^,^^51^

### Age

Traumatic tears are associated with younger patients, a finding supported by several studies^6^^,^^9^^,^^52^ and confirmed by the present research. Patients from this age range present the tendency to prematurely resume usual activity and may consequently show less compliance regarding postoperative immobilization.^53^ Lack of compliance is more prone to jeopardize anterior RCRs, which are more prevalent in traumatic tears. Indeed, subscapularis elongation during postoperative mobilization may display a maximal excursion of up to 122%, as opposed to the supraspinatus which is systematically shortened when mobilized.^36^ Furthermore, younger categories of age are more demanding and may thus report lower subjective scores such as SSV.^54^

### Localization of the Tear

Only 14% of the total strength of the rotator cuff is generated by the supraspinatus,^55^ as opposed to the 53% provided by the subscapularis tendon. Therefore, deficiency of the latter, which is preponderant in traumatic settings, creates functional impairment by means of imbalance and deficit of anterior forward flexion.^27^

### Prevalence

Traumatic tears were considered to be relatively uncommon, with a prevalence ranging from 8.4% to 17.7% in the current literature.^4^^,^^8^^,^^56^^,^^57^ The present study illustrates a greater occurrence of traumatic cases, representing 50% of the cohort. The prevalence rather depends on the type of surgical activity, insurance coverage, and enrollment. A recent national study highlighted these discrepancies among various institutions belonging to the same health care system.^13^

### Surgical Timing

The onset of symptoms before surgery significantly differed between groups, with an average of 3 months in the traumatic group compared with 15 months in that of the degenerative (*P* < .001). Patients who benefit from surgery within 3 weeks may expect the best functional outcomes,^4^^,^^51^ in contrast to patients who experience functional decline by waiting 4 months after an injury to receive surgery.^51^ In this study, only 28% of patients were operated on within 3 weeks, a delay that may have compromised results in the traumatic group.

Traumatic RCRs are rather common and tend to involve more of the subscapularis as well as younger patients and men. These tears also present more retraction. They are not simply an acute failure of a degenerative tendon but represent a distinct pathologic entity. As previously highlighted by Amini et al.,^1^ the findings of this study also support the current practice of treating traumatic tears differently from degenerative ones. Radiologic signs of healing and satisfactory clinical outcomes seem to be achieved in both groups regardless of time between presentation and surgery, changing the paradigm in which traumatic tears evolve more favorably than degenerative RCRs. Tendon fraying and shortening, muscle edema and fibrosis, younger age, anterior tears, and precipitated surgery in traumatic situations are possible explanations.

The strength of this study is a large, homogeneous cohort with both pre- and postoperative radiologic and clinical evaluations. The proper distinction between traumatic and degenerative tears is important, using strict and objective criteria, contrary to vague definitions of previous publications. Only shoulders with a low level of fatty infiltration were included to avoid the dilemma of irreparable injuries. One surgeon performed each surgery with similar types of repairs in each group.

### Limitations

This study is not without limitations. Implementation of associated procedures was not standardized between the 2 groups; the frequency of acromioplasty, tenotomy of the long head of the biceps, and clavicular resection were greater in patients with degenerative RCRs. However, we performed a multivariate regression to consider such differences. The postoperative rehabilitation protocol was different for each RCR, which may have led to a disparity between groups. The number of superior RCRs for which immobilization was not proposed, however, was similar between groups; 37.9% did not wear slings in the traumatic group versus 38.1% in the degenerative one (*P* = 1.000). Lastly, ultrasound scans had not been performed by an independent examiner.

## Conclusions

Traumatic cases were frequent, involved younger patients, more frequently affected the anterior rotator cuff, and were associated with more severe tendon retraction. Traumatic and degenerative RCRs lead to comparable clinical and radiologic results.

## Disclosure

The authors declare the following financial interests/personal relationships which may be considered as potential competing interests: FORE (Foundation for Research and Teaching in Orthopedics, Sports Medicine, Trauma, and Imaging in the Musculoskeletal System) Grant # FORE 2023-49. A.L. reports grants from Arthrex, during the conduct of the study; personal fees from Stryker, from null, outside the submitted work; and He is the (co)founder of FORE, Med4Cast, and BeeMed. P.C. reports personal fees from Arthrex, during the conduct of the study; and personal fees from Stryker and Fx Solution, outside the submitted work. All other authors (A.G.-A., E.A.V.R., H.B., J.Z., A.G.) declare that they have no known competing financial interests or personal relationships that could have appeared to influence the work reported in this paper. Full ICMJE author disclosure forms are available for this article online, as supplementary material.

## Supplementary Data

ICMJE author disclosure forms
